# Quantitative microscopy uncovers ploidy changes during mitosis in live *Drosophila* embryos and their effect on nuclear size

**DOI:** 10.1242/bio.022079

**Published:** 2017-01-20

**Authors:** Wee Choo Puah, Rambabu Chinta, Martin Wasser

**Affiliations:** Imaging Informatics Division, Bioinformatics Institute (BII), Agency for Science, Technology and Research (A*STAR), Singapore 138671, Republic of Singapore

**Keywords:** Drosophila, Live imaging, Maternal haploid, Spartan, Quantitative microscopy, Mitosis

## Abstract

Time-lapse microscopy is a powerful tool to investigate cellular and developmental dynamics. In *Drosophila melanogaster*, it can be used to study division cycles in embryogenesis. To obtain quantitative information from 3D time-lapse data and track proliferating nuclei from the syncytial stage until gastrulation, we developed an image analysis pipeline consisting of nuclear segmentation, tracking, annotation and quantification. Image analysis of *maternal-haploid* (*mh*) embryos revealed that a fraction of haploid syncytial nuclei fused to give rise to nuclei of higher ploidy (2n, 3n, 4n). Moreover, nuclear densities in *mh* embryos at the mid-blastula transition varied over threefold. By tracking synchronized nuclei of different karyotypes side-by-side, we show that DNA content determines nuclear growth rate and size in early interphase, while the nuclear to cytoplasmic ratio constrains nuclear growth during late interphase. *mh* encodes the *Drosophila* ortholog of human Spartan, a protein involved in DNA damage tolerance. To explore the link between *mh* and chromosome instability, we fluorescently tagged Mh protein to study its subcellular localization. We show Mh-mKO2 localizes to nuclear speckles that increase in numbers as nuclei expand in interphase. In summary, quantitative microscopy can provide new insights into well-studied genes and biological processes.

## Abstract (precis)

**Summary:** A new 3D time-lapse microscopy image analysis pipeline consisting of nuclear segmentation, tracking, annotation and quantification revealed karyotype changes in *Drosophila* embryos.

## INTRODUCTION

Time-lapse microscopy of living tissues and cells in three dimensions provides new insights into the dynamics of biological systems and promises the discovery of new gene functions and biomolecular mechanisms. However, transforming multi-dimensional images into valuable information requires smart software for data quantification and visualization. In developmental biology, dedicated software tools have been developed for cell lineage analysis using fluorescently labeled nuclei in models like *Drosophila* (Amat et al., 2014; Minden et al., 1989), *Caenorhabditis* *elegans* (Bao et al., 2006; Long et al., 2009) and zebrafish (Keller et al., 2008; Lou et al., 2011). In the cell cycle field, automated image analysis systems have been developed for genome wide image-based screens in culture cells for genes involved in cell divisions (Neumann et al., 2010). Less research has been directed at developing methods for the study of cell divisions in multi-cellular organisms such as *Drosophila* (Chinta and Wasser, 2012; Yau and Wakefield, 2007). Most image analysis pipelines can be divided into four major steps: segmentation, tracking, annotation and statistical analysis. Image segmentation detects regions of interest as areas (2D) or surfaces (3D) that enclose biological objects, such as cells or nuclei (Coelho et al., 2009; Gul-Mohammed et al., 2014; Li et al., 2007). In time series microscopy, tracking establishes associations between objects in different frames and is required to measure temporal features and reconstruct cell lineages (Meijering et al., 2009). Annotation assigns biological meaning to detected objects. For high-throughput analysis, machine learning is essential for automated phenotypic classification (Sommer and Gerlich, 2013). Besides customized software, many of the image analysis steps can be performed by generic open source or commercial packages (Eliceiri et al., 2012).

*Drosophila* embryogenesis is a useful model to study the cell cycle in the context of a developing multi-cellular organism (Foe and Alberts, 1983; Garcia et al., 2007). Genetics allow the analysis of homologues of human genes that are relevant for diseases like cancer (Bier, 2005; Halder and Mills, 2011). Upon fertilization, the two haploid gametes fuse to give rise to diploid zygote. Nuclei divide synchronously 13 times in a common cytoplasm or syncytium. After completion of the syncytial blastoderm, nuclei are engulfed by a plasma membrane to generate cells (Lecuit and Wieschaus, 2000). Once cellularization is complete, cells, concurrent with gastrulation, resume divisions in so-called mitotic domains (Foe and Alberts, 1983). The transparency of embryos enables live cell imaging using fluorescent proteins. While early studies relied on the injection of fluorescently labeled antibodies or proteins (Minden et al., 1989; Warn et al., 1987), modern approaches are based on readily available genetically encoded fluorescent fusions such as histone tagged to green fluorescent protein (GFP) or its variants (Shaner et al., 2005). In *Drosophila*, haploidy can result from the failure of pronuclear fusion during fertilization. The mutation *maternal haploid* (*mh*) was isolated in a screen for female sterile mutations (Gans et al., 1975). *mh* leads to gynogenetic development due to the elimination of the male pronucleus (Santamaria, 1983) which has been proposed to be caused by defects in sperm chromatin remodeling, DNA replication or chromosome condensation (Loppin et al., 2001). The *mh* gene encodes the fly ortholog of the recently identified human Spartan protein, a conserved regulator of DNA damage tolerance (Delabaere et al., 2014).

The discovery that the sizes of cytoplasm and nucleus are positively correlated dates back over a century. However, little is known about the mechanisms that establish and maintain the nuclear-cytoplasmic (NC) ratio (Jevtić et al., 2014; Webster et al., 2009). Understanding the mechanism is relevant because NC ratio is correlated with cancer and ageing. The nucleoskeletal theory proposes that the amount and compaction of DNA influences the size of the nucleus which in turn determines the size of the cell (Cavalier-Smith, 1978). Conflicting with this idea are more recent data obtained in fission and budding yeast that suggest that cell size determines nuclear size (Jorgensen et al., 2007; Neumann and Nurse, 2007). Studies in *Drosophila* and *Xenopus* demonstrated the role of the nuclear envelope in controlling nuclear size (Brandt et al., 2006; Levy and Heald, 2010).

Previously, we developed a 3D level set-based segmentation method for interphase nuclei and mitotic chromosomes in *Drosophila* embryogenesis (Chinta and Wasser, 2012). Here, we report the extension of this work to detect and characterize phenotypic effects of cell cycle defects in selected lineages. To validate our approach in studying karyotype changes resulting from mitotic division defects, we applied our method to time-lapse data of *mh* embryos. We discovered that haploid nuclei in *mh* embryos can collide during the syncytial blastoderm to give rise to diploid and polyploid nuclei that persist until gastrulation and resume mitosis. Being able to track synchronized nuclei of different karyotypes side-by-side in the same embryo allowed us to investigate the contributions of DNA content and NC ratio to nuclear volume. In early interphase after exit from mitosis, nuclear growth is proportional to DNA content, while in late interphase, the nuclear density imposes constraints on volume expansion in the later stages of interphase. In addition, we demonstrate that fluorescently tagged Mh-protein localizes to nuclear speckles and is able to rescue female sterility of the *mh*^1^ mutation.

## RESULTS

### Image analysis pipeline for the study of division cycles in live *Drosophila* embryos

**Fig. 1.**
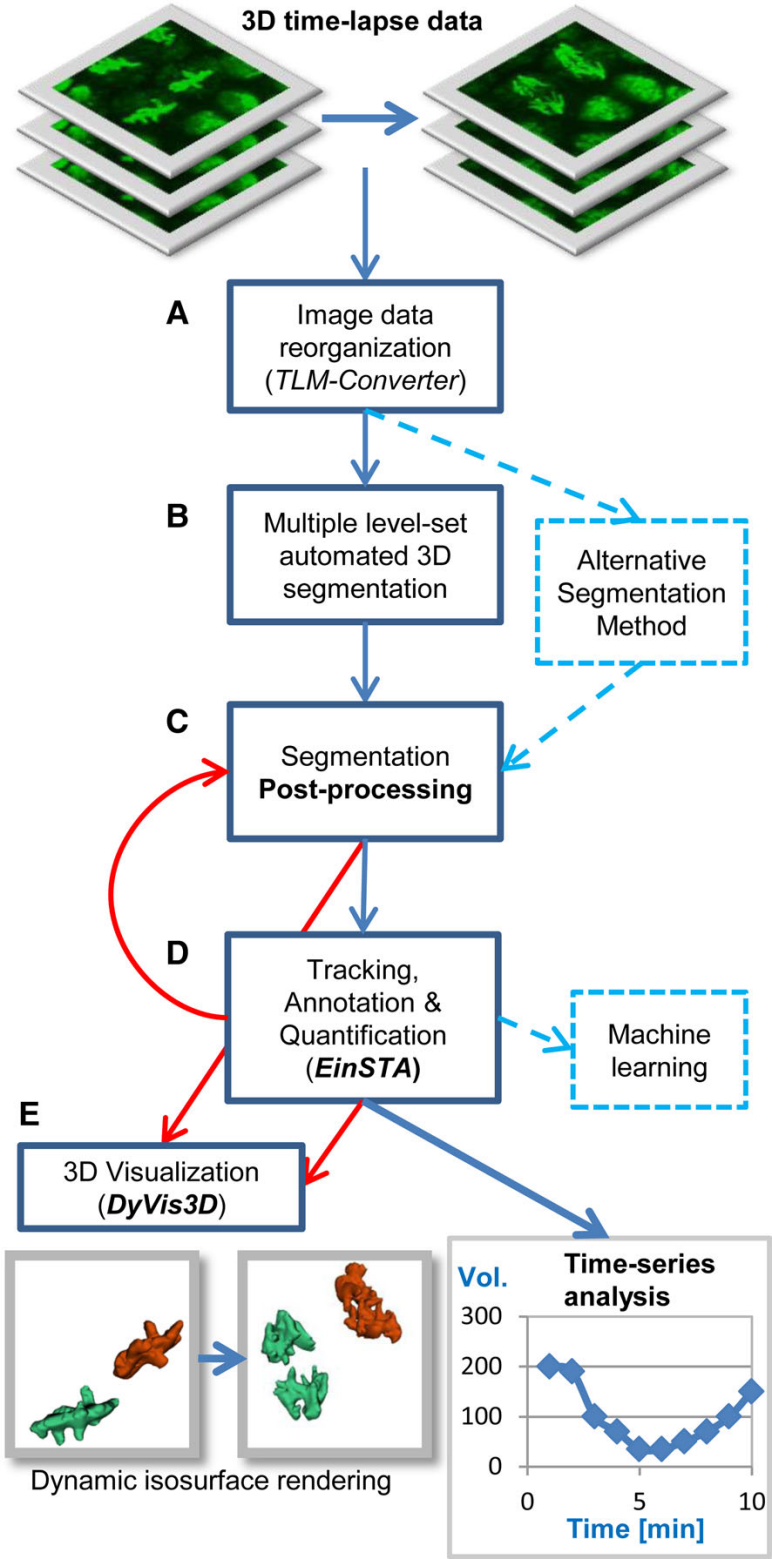
**Image analysis workflow for 3D time-lapse microscopy.** 3D time-lapse datasets were processed using a pipeline of five standalone software modules. (A) The TLM-Converter fragmented the 3D image data to facilitate downstream processing tasks, such as image segmentation. For this study, datasets were split into portions of 10 time-points. (B) Automated 3D segmentation detected nuclei and saved their features and surfaces. (C) The optional post-processing module split merged ROIs and calculated additional 3D features. (D) The einSTA (editing and integration of Segmentation, Tracking and Annotation) tool performed tracking and time-series analysis. Objects can be manually labeled and exported for machine learning tasks. (E) The dynamic visualization module *DyVis3D* rendered and animated selected tracks in 3D.

To track nuclei in early *Drosophila*, we acquired 3D time-lapse images of embryos expressing the live chromosome reporter Histone H2Av-GFP, hereafter referred to as histone-GFP (Clarkson and Saint, 1999). Image recording was carried out using a Zeiss 5 Live confocal laser scanning microscope from the syncytial blastoderm until gastrulation for up to 4 h at 55 to 60 s intervals. To measure and visualize the morphological changes of interphase nuclei and mitotic chromosomes, we built a semi-automated image analysis system that comprises five standalone modules (Fig. 1). (A) A batch file converter (TLM-converter) fragments large multi-dimensional datasets for easier downstream processing into smaller portions (Puah et al., 2011). (B) The batch 3D image segmentation tool (Chinta and Wasser, 2012) detects the regions of interest (ROIs) that correspond to nuclei. (C) The optional post-processing module extracts additional data from the primary segmentation outputs. (D) The einSTA (editing and integration of Segmentation, Tracking and Annotation) tool performs tracking and quantification of dynamic features. (E) The DyVis3D module visualizes selected lineages by iso-surface volume rendering. While steps B and C use images as inputs to detect ROIs and compute their features, steps D and E use ROIs as inputs to perform tracking, quantification of cellular dynamics and 3D visualization.

The 3D nuclear segmentation method takes advantage of multiple level sets to adapt to local variations in histone-GFP intensity due to cell cycle dependent variations in chromatin compaction (Chinta and Wasser, 2012). For each detected nucleus, the tool produces a primary segmentation output consisting of a 3D surface (represented as a stack of 2D contours), a silhouette contour of the 3D shape, an optimal intensity threshold, and a set of intensity and shape features (Fig. S1). einSTA performs time-series analysis of detected ROIs which are represented by silhouette contours and centroids that are projected onto maximum intensity projections (MIP) of image stacks. The visual cues help to spot segmentation errors such as merged or fragmented ROIs (Fig. S2). In proliferating tissues, the size, shape, texture and brightness of biological structures such as chromosomes can vary depending cell cycle phase or genotype. To improve robustness of image analysis towards this phenotypic heterogeneity, we introduced the concept of composite segmentation. Each frame in a time-series dataset is associated with one or more segmentation layers (Fig. S2A and Fig. S2B), each of which represents a set of ROIs that was produced by a different segmentation method and/or set of parameters applied to the same image stack. An additional composite layer is then created to be populated with combinations of ROIs derived from different segmentation layers. In our case study, we initialized the composite layer with all ROIs detected by the MLS method using a parameter set optimized for a set of ground truth images. The second layer was produced by applying a shape-split operation to the ROIs of the primary layer (Fig. S3). Incorrectly segmented objects in the composite layer were removed and substituted in an interactive fashion with objects from the second layer.

Tracking creates temporal associations between ROIs of adjacent frames and is required to determine dynamic features. We applied a bilateral nearest neighbor (NN) search to find assignments between ROI centroids of subsequent frames. This automated step was followed by manual correction of assignment errors. Based on 27818 ground truth assignments with a mean displacement of 1.41 µm±1.61 µm (median 0.90) in 121 pairs of frames in an embryo imaged from nuclear cycle 12 until cell cycles in gastrulation, recall and precision rates of the NN search were estimated as 94.6% and 91.8%, respectively (Fig. S4). Once the accuracy of selected tracks was verified, time-series data such as volume and intensity changes were exported for further statistical analysis (see next chapters).

### Fusions of nuclei in *mh*^1^ syncytial embryos lead to karyotype changes

**Fig. 2.**
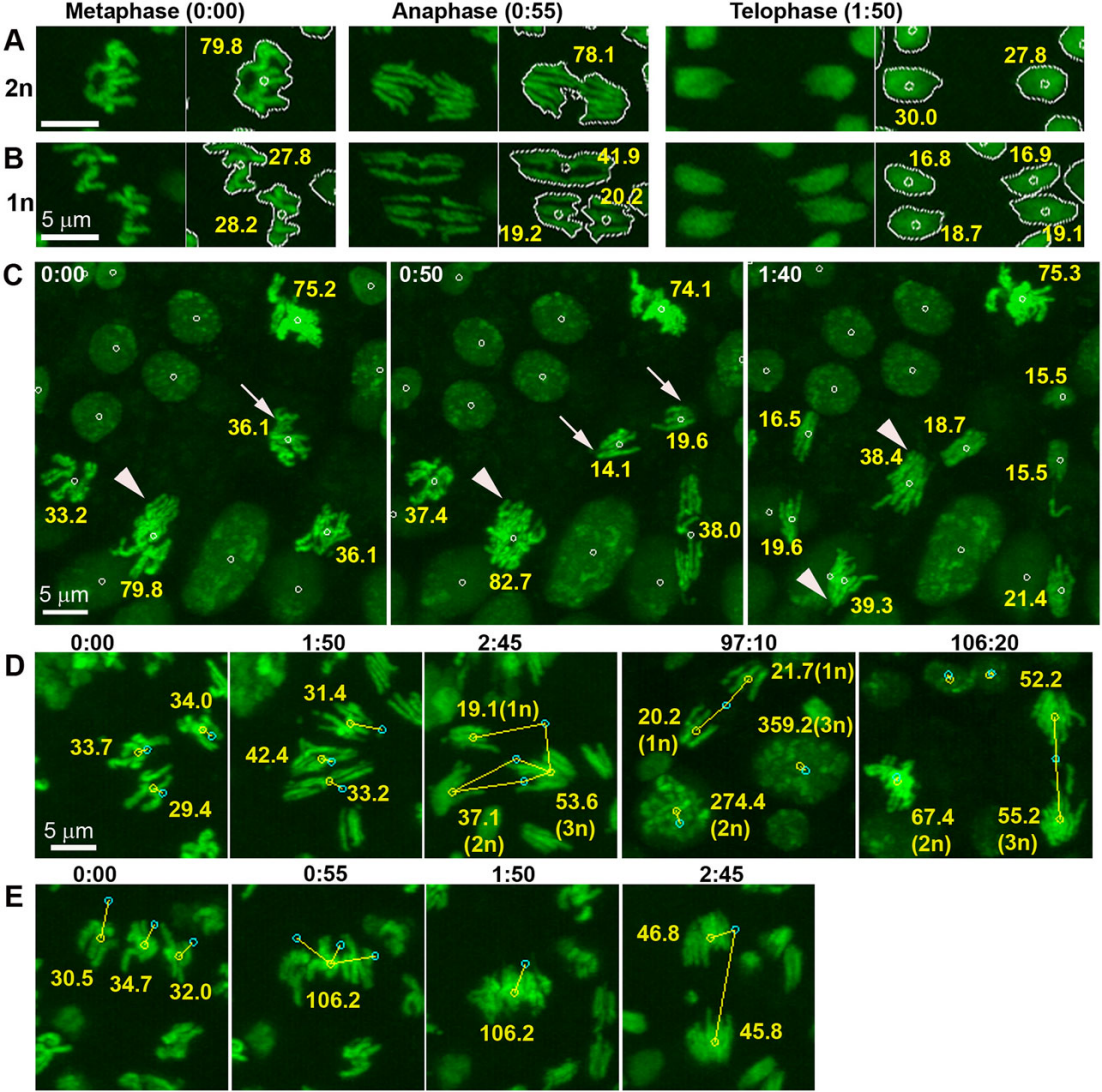
**Collisions of mitotic chromosomes in *maternal haploid* (*mh*^1^) syncytial blastoderm embryos lead to changes in ploidy.** (A) Diploid (2n) karyotypes of control can be visually distinguished from (B) haploid (1n) karyotypes of *mh*^1^ embryos in mitotic chromosomes labeled with histone-GFP (green). Time stamps in min:sec. Left panels show maximum intensity projections of live mitotic chromosomes that were recorded at 55 s intervals in metaphase, anaphase and telophase, while the right panels show the same images with three annotations obtained from nuclear segmentation. The silhouette contours around the segmented nuclei and the centroids (small circle) are drawn in white. The volume measurements (µm^3^, yellow) are proportional to DNA content. (C) *In vivo* imaging during gastrulation revealed that *mh*^1^ embryos contained nuclei of different ploidy. Volume measurements indicate that mutant embryos contained haploid (arrow) as well as diploid nuclei (arrow heads). (D,E) Time-lapse image analysis revealed that mitotic chromosomes in *mh*^1^ syncytial blastoderm embryos collided during metaphase (D) or anaphase (E), giving rise to changes in ploidy. Yellow lines connect centroids of previous (cyan) and current (yellow) frame. (D) At 1:50, three haploid nuclei underwent anaphase. At 2:45, sister chromatids collided to create one haploid, one diploid and one triploid instead of six haploid daughter nuclei. All three daughters persisted into post-cellular blastoderm to resume non-synchronized mitotic divisions. The haploid cell divided at 97:10 and the triploid cell at 106:20. (E) In this sequence, chromosome of three haploid nuclei (0:00) fused during metaphase (0:55). The subsequent anaphase (2:45) divided the chromatids into two triploid instead of 6 haploid daughter nuclei.

Cell division defects can lead to alterations of chromosome number. To test if our analysis system could detect variations in ploidy, we decided to compare *maternal haploid* (*mh*^1^) with diploid wild-type embryos. Consistent with haploidy, histone-GFP labeled mitotic chromosomes of *mh*^1^ embryos occupied a discernibly smaller regions than in diploid embryos (Fig. 2A,B) and the presence of 5 chromosome arms in haploids (one arm for the X, two arms each 2nd and 3rd) instead of 10 in diploids could often be confirmed visually in images of anaphase nuclei (Fig. S5). Volume measurements showed that mitotic chromosomes occupied approximately half the space in haploid embryos compared to their diploid counterparts (Fig. 2A,B). Furthermore, chromosomal volume statistics of *mh*^1^ and control embryos showed that measurements during metaphase and anaphase were proportional to DNA content (Fig. S6A). To assess the influence of variations in histone-GFP reporter gene expression on volume measurements, we analyzed the fluorescence intensity of detected objects. We noticed that mean histone-GFP intensity increased consistently between subsequent nuclear cycles (29.5%±5.7%, *P*=0.000, mean µ>0, *n*=8). In contrast, we did not observe a corresponding increase in measured volumes (−5.6%±10.6%, *P*=0.179, µ≠0), indicating that our image segmentation method was robust towards variations in fluorescence intensity (Fig. S6B).

Unexpectedly, images of some post-blastoderm *mh*^1^ embryos showed nuclei of variable sizes and chromosome numbers, indicating a mixture of karyotypes (Fig. 2C). Volume measurements of mitotic chromosomes in metaphase, anaphase and telophase suggested that a fraction of cells contained between two to four times more DNA than haploid cells in the same embryo. To understand the diversity in karyotypes, that had also been reported in previous studies (Loppin et al., 2001; Santamaría and Gans, 1980), we traced individual nuclei from the post-blastoderm (gastrula) back to the syncytial blastoderm stage using the einSTA tool. Time-lapse image analysis revealed that increased ploidy arose from collisions of mitotic chromosomes during the syncytial blastoderm (Fig. 2D,E; Movies 1, 2 and 3). Collisions led to volume changes proportional to chromosome numbers. Diploid nuclei in mutant *mh*^1^ embryos gave rise to mitotic chromosomes that occupied twice the volumes of haploid chromosome sets in the same embryo in metaphase and anaphase, and comparable volumes to diploid chromosome sets in control embryos. 3D visualization of tracks in 3D helped to rule out segmentation errors as explanations for karyotype changes (Movie 3). All observed collisions (*n*=87) happened during metaphase (25%) or anaphase (75%) of the syncytial blastoderm (Fig. 2D,E). Chromosome collisions were rare events. The estimated proportion of nuclei per nuclear cycle involved in collisions was 4.5%±3.8% (1399 nuclei in 9 nuclear cycles) (Fig. S7). As previously reported, *mh*^1^ embryos undergo 14 instead of 13 nuclear divisions prior to cellularization (Edgar et al., 1986). Hence it seemed conceivable that chromosome fusions could arise from the increased density of nuclei during nuclear cycle 14. However, we detected several fusions during the earlier nuclear cycles 12 and 13, indicating that physical crowding in the periphery of embryos was unlikely to be the cause of nuclear collisions (Fig. S7). We did not observe any chromosome collisions during the syncytial blastoderm of wild-type embryos (1015 nuclei during NC11-13 of 3 embryos). Collisions in anaphase did not involve daughter nuclei from the same mother nucleus, arguing against defects in chromosome segregation as an explanation for karyotype changes. Moreover, we never observed fusions of interphase nuclei during syncytial blastoderm or later in embryogenesis after cellularization. The majority of cases involved two haploid nuclei merging to become diploid. In one case, three haploid genomes gave rise to one haploid, diploid and triploid nucleus by colliding in anaphase (Fig. 2D). In another case, we saw a simultaneous fusion of the three haploid genomes in metaphase (Fig. 2E). Our tracking data did not provide any evidence that diploidy in haploid embryos resulted from endoreplication during the cellular blastoderm since we did not to detect any track that changed its ploidy between the last syncytial and the first non-synchronized division in gastrulation. Another explanation of nuclear collision could be that haploidy could make chromosomes more prone to collisions. However, we also observed diploid nuclei in *mh*^1^ embryos that fused to become tetraploid (Movies 2 and 3). In summary, the combination of 3D time-lapse imaging and image analysis enabled the discovery that chromosome collision lead to karyotype changes in *mh*^1^ embryos.

### Diversity of nuclear densities in *mh* embryos at mid-blastula transition

**Fig. 3.**
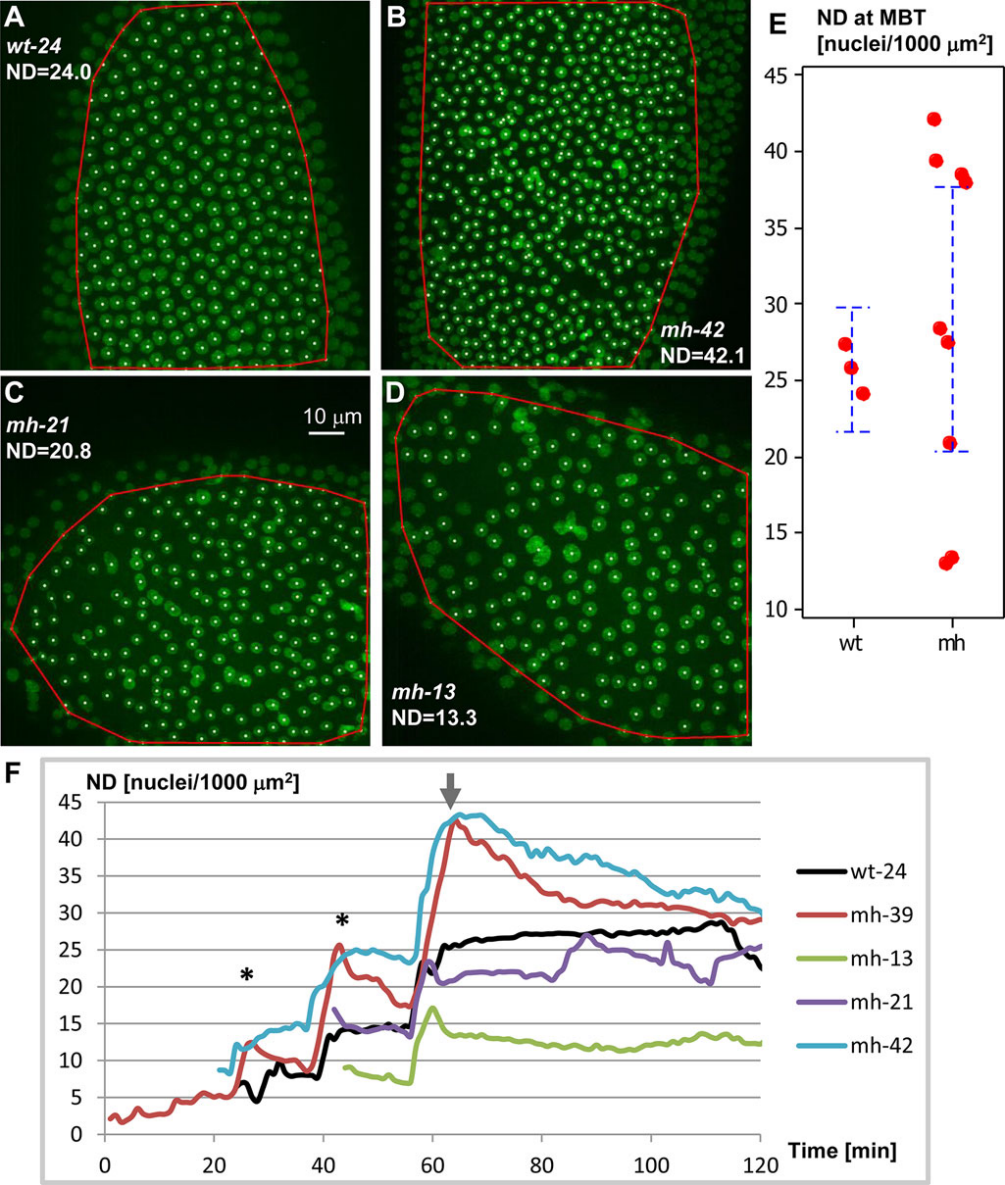
**Variations of nuclear density at midblastula transition (MBT) of *mh*^1^ embryos.** Nuclear density or ND (#nuclei/1000 µm^2^) was used to monitor proliferation and depletion of nuclei. ND was calculated by dividing the number of detected nuclei (centroids marked by dots) with the area of the convex hull (red curve). (A-D) One control (wt-24) and three *mh*^1^ embryos after completion of the final synchronized mitosis. Compared to 13 nuclear cycles in wild-type (A), the majority of *mh*^1^ embryos undergo 14 nuclear cycles (B) that lead to higher ND values. A smaller proportion of *mh*^1^ embryos, enter MBT with NDs similar to (C) or lower (D) than control. (E) *mh*^1^ embryos show a broader ND distribution at the onset of MBT. Error bars indicate 95% confidence intervals (CI). (F) Plotting ND against time reveals proliferation and depletion of surface nuclei in one wild-type and four *mh*^1^ embryos (see Table 1) during the last 1-3 synchronous nuclear division cycles and subsequent cellularization. All plots are aligned with respect to the last synchronized mitosis (arrow). Anaphase (*) of the synchronous nuclear cycles (NC) is accompanied by a doubling of ND. Consistent with one additional NC in *mh*^1^ mutants, the embryos mh-39 and mh-42 showed higher ND readouts at MBT. Due to elimination of cortical nuclei, ND values declined after anaphase, while remaining constant in the control embryo wt-24. A small proportion of *mh*^1^ embryos entered MBT at nuclear densities similar to (mh-21) or lower (mh-13) compared to controls.

Mitotic defects during the syncytial blastoderm stage are known to lead to an elimination of surface nuclei (Philp et al., 1993). To quantify nuclear proliferation and elimination, we implemented a method to estimate the density of surface nuclei (Fig. 3A-D). The method computes a convex hull using the x-y components of nuclear centroids as inputs. Nuclear density (ND) was determined as the number of nuclei divided by their enclosing hull area (#nuclei/1000 µm^2^). We analyzed the time-lapse datasets of three control and nine *mh*^1^ embryos labeled with histone-GFP. ND values determined 5.5 min after start of anaphase of the final synchronized metaphase showed a greater variability in *mh*^1^ (mean=28.7±11.7; ranging from 12.2 to 42.1) than in control embryos (26.5±1.8; ranging from 24.5 to 27.6) (Fig. 3E). Consistent with previous reports that *mh*^1^ embryos undergo an additional nuclear cycle before entering mid-blastula transition (MBT, here defined at as the period following the last synchronized division cycle), four *mh*^1^ embryos showed up to 60% higher nuclear densities than controls after the exit from the last syncytial mitosis. Time series plots revealed that ND decreased during the cellular blastoderm (Fig. 3F). As a result, after 40 min, the increased ND over controls narrowed to less than 30%. The decreasing ND was reflected by an elimination of surface nuclei that could be observed in the time-lapse images of live *mh*^1^ embryos (Movie 1). Unexpectedly, we encountered a second group of *mh* embryos that entered MBT with nuclear densities lower than (three embryos) or similar to (two embryos) controls (Fig. 3E,F). In summary, the quantification of nuclear density and its developmental dynamics added new phenotypic insights about *mh*^1^ mutants.

### Control of interphase nuclear volume during the cellular blastoderm

**Fig. 4.**
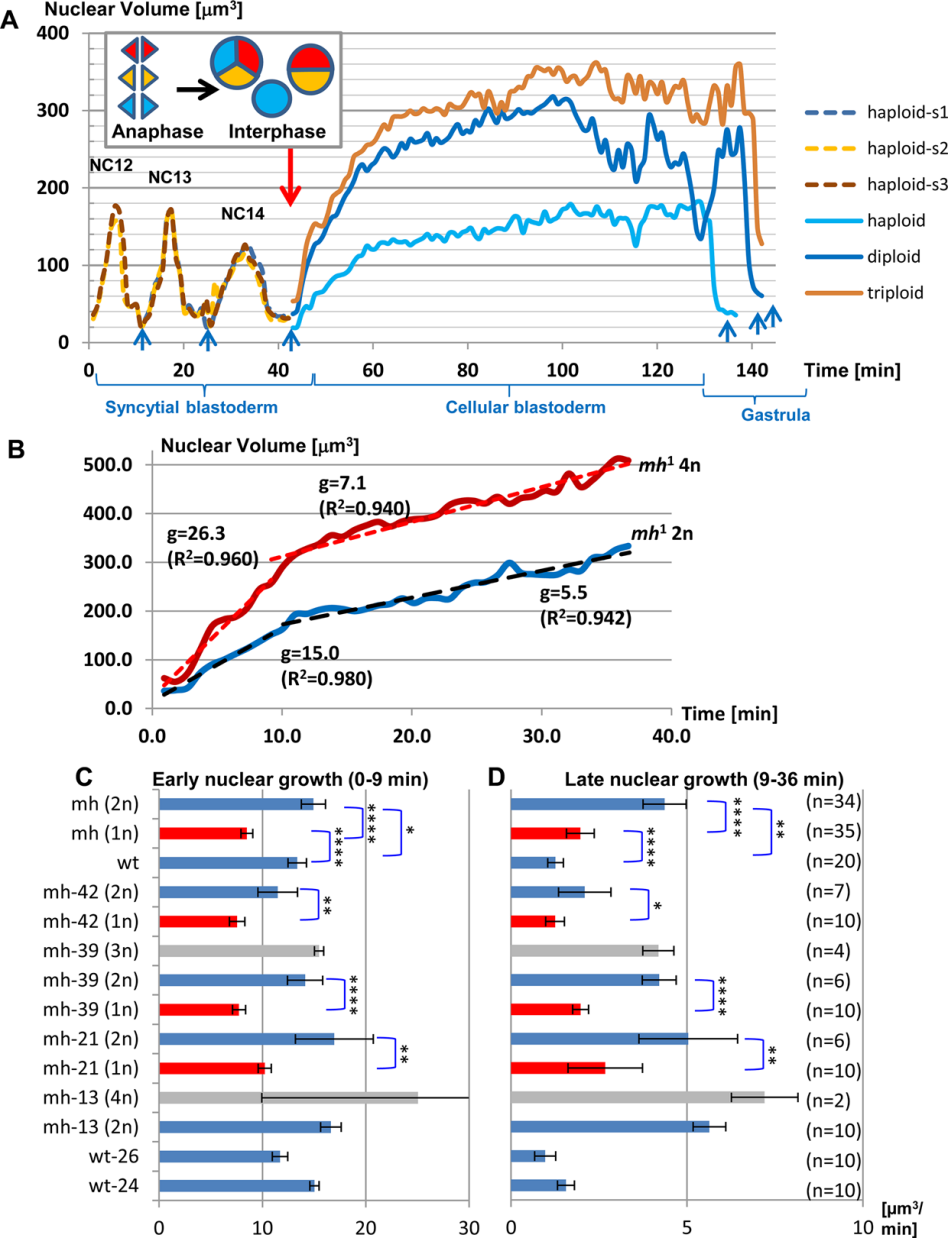
**The sizes of interphase nuclei and their growth rates positively correlate with DNA content.** (A) Mitotic chromosomes of three syncytial haploid nuclei (s1-s3) collided during anaphase of NC14 (inset, red arrow) to give rise to synchronized daughter nuclei of different ploides (1n, 2n, 3n). See Fig. 2D and Movie 1. The line plots indicate that nuclear growth and size positively correlate with DNA content. Blue arrows indicate anaphase, the peaks during syncytial blastoderm correspond to late interphase of the haploid nuclei from NC12-14. (B) To quantify nuclear volume expansion following the last synchronous mitosis, we divided interphase into a rapid early phase of 7 min and a late slow growth phase of 37 min and determined linear regression. This plot compares the volume changes of diploid (2n) and tetraploid (4n) nuclei after exit from mitosis in embryo mh-13. Nuclear growth rates *g* derived from linear regression are positively correlated with ploidy. R^2^ values in brackets. (C,D) Bar charts compare early and late growth rates (mean±95% CI) of two control and four *mh*^1^ embryos (see Table S1). Within embryos, nuclear growth rates scale with DNA, i.e. diploid grow significantly faster than haploid nuclei in the same syncytium. *t*-tests *P≤0.05, **P≤0.01, ****P≤0.0001.

**Fig. 5.**
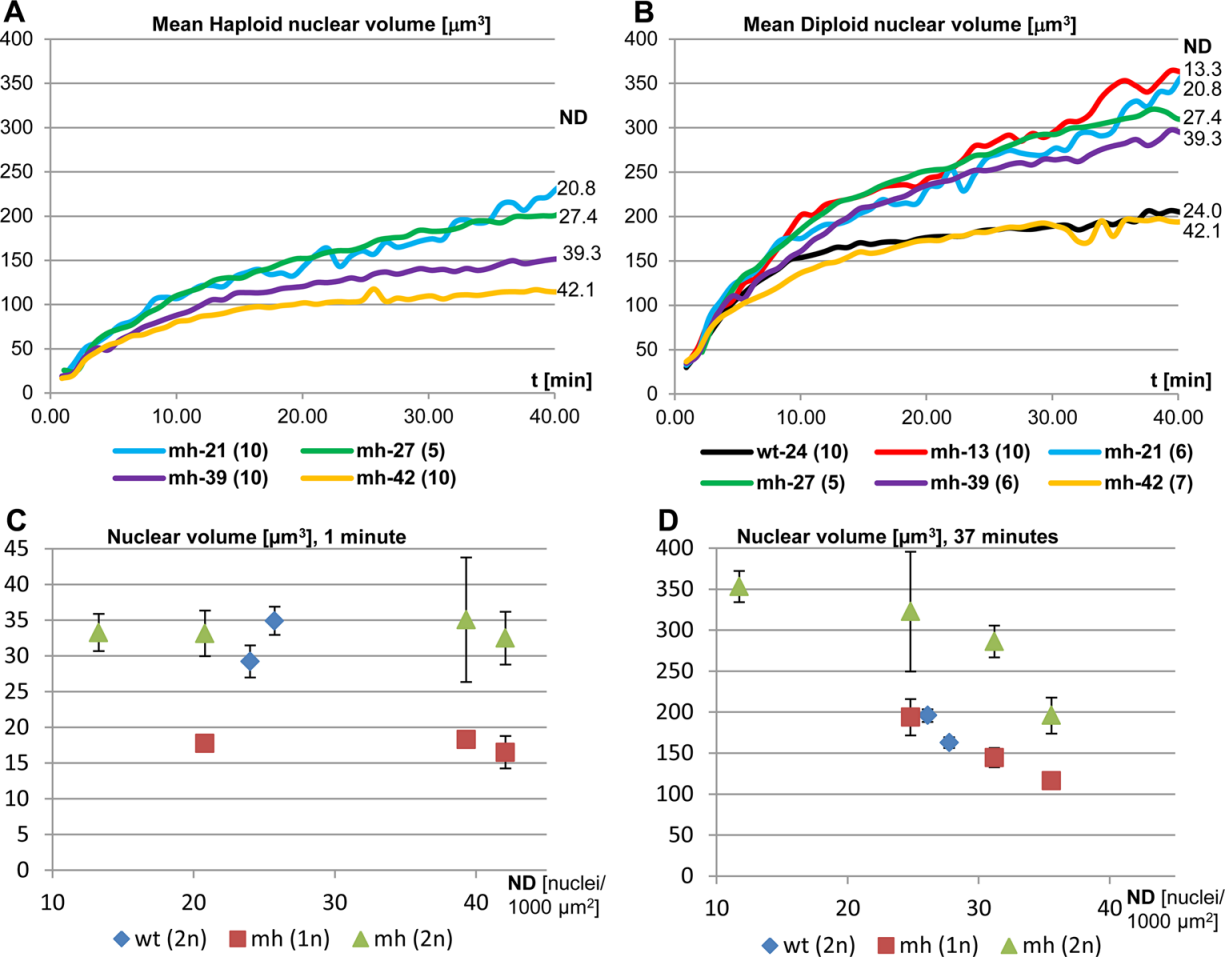
**The sizes of interphase nuclei are inversely correlated with nuclear density.** The volumes of (A) haploid and (B) diploid nuclei and their growth rates differ between embryos and are inversely correlated with nuclear density. The scatter plots show mean volume measurements for the initial 40 min after exit from the last synchronized mitosis. Numbers next to the right ends of the lines indicate ND at the beginning of MBT. (Sample sizes in brackets) (C,D) Nuclear volume depends on both DNA content and ND. Scatter plots show the relationship between ND and nuclear volumes (mean±95% CI) at anaphase of the last synchronized mitosis (C) and during interphase 37 min later (D). Diploid nuclei grow faster than haploid ones in the same neighborhood. Sample size: control embryos wt(2n) were wt-24 and wt-26 (*n*=10), *mh*^1^ embryos (*n* haploid, *n* diploid nuclei) were mh-13 (0,10), mh-21 (10,6), mh-39 (10,6) and mh-42 (10,7).

Published data contain conflicting theories on the factors that determine the size of the nucleus. On one hand, the nucleoskeletal theory proposes that the amount of DNA is the major determining factor (Cavalier-Smith, 1978), while on the other hand, experimental data in yeast suggest that cytoplasmic size dictates the volume of the nucleus independent of ploidy (Jorgensen et al., 2007; Neumann and Nurse, 2007). The diversity in nuclear densities and karyotypes at the onset of MBT in *mh*^1^ embryos opened the opportunity to analyze the influence of DNA content and NC-ratio on nuclear growth rate and volume. We focused our analysis on cellularization which begins after the exit from the last syncytial mitosis and takes approximately one hour to complete (Lecuit, 2004). Time-series plots comparing the volumes of individual nuclei of different karyotypes in the same embryo showed that volumes were proportional to the amount of DNA (Fig. 4A). In addition, we noticed two distinct phases of nuclear growth during the cellular blastoderm stage, an initial rapid growth lasting 8-10 min (phase 1) and a subsequent slow growth phase (phase 2) that lasts until the next mitosis (Fig. 4B). To estimate and compare nuclear growth rates within and between embryos, we determined the slopes (µm^3^/min) of the linear regressions (volume versus time) that were computed for the first 10 frames (9.1 min) after anaphase and the subsequent 30 frames (27.5 min) (Fig. 4B). We performed the quantification of 89 nuclear tracks derived from seven embryos (5 *mh*^1^ and two control) that were recorded from the syncytial nuclear divisions until the post-blastoderm cell divisions (Table S1). The mean R^2^ values of the tracks were 0.941±0.046 and 0.850±0.175 for phases 1 and 2, respectively, indicating that the linear growth model represented a good approximation. Ploidies of the analyzed nuclei ranged from 1 to 4, while nuclear densities after exit from the last syncytial mitosis ranged from 12.2 to 42.1 nuclei per 1000 µm^2^. The deceleration in nuclear growth between early phase 1 and late phase 2 ranged from 2.9 to 9.6. When comparing synchronized nuclei of different karyotypes within the same embryos, growth rates (Fig. 4C,D) and volumes (Fig. S8) were always proportional to DNA content throughout the cellular blastoderm stage, supporting the nucleoskeletal theory. Meanwhile, we noticed that sizes of nuclei of the same ploidy showed increasing divergence between embryos as interphase progressed (Fig. 5A,B). Plotting the mean volumes versus ND of haploid and diploid nuclei at anaphase (Fig. 5C) and 36 min later in interphase (Fig. 5D) further helped to visualize the negative effect of ND on nuclear size.

Interphase of the nuclear cycles 1-13 only consists of S-phase and lacks the gap phases G1 and G2, while interphase of the cellular blastoderm comprises S- and G2-phases (Farrell and O'Farrell, 2014). To examine the influence of G2 on nuclear growth, we quantified nuclear size changes during the last two synchronized cycles of control and *mh*^1^ embryos. During the penultimate nuclear cycles, we observed that a linear and rapid nuclear growth was followed by DNA condensation (Fig. S9A), suggesting that the rapid nuclear growth coincides with DNA replication and slow growth with G2. The comparison of nuclei of different ploidy in *mh*^1^ embryos confirmed that, in the syncytial blastoderm, nuclear size (Fig. S9B) and growth (Fig. S9C) scaled with ploidy.

To explore if a limited pool of maternally supplied nuclear assembly factors may impose a collective constraint on nuclear growth, we determined the sum of nuclear volumes relative to the area they occupy on the embryonic surface. We refer to the cumulative nuclear volume as the volume-to-hull ratio (VHR). Time series plots indicate that VHR values of different embryos converged to a narrow range from 4.2 to 5.9 (5.3±0.6) despite the diversity of karyotypes and nuclear densities (28.4±9.4), supporting the idea that the NC-ratio limits nuclear growth (Fig. S10). The collective volume analysis does not require tracking of individual nuclei and is tolerant to segmentation errors like fusion or splitting of ROIs, thus offering a more efficient way to analyze time-lapse data. In summary, we demonstrate that nuclear growth rates are determined by both DNA content and the amount of cytoplasm available for each nucleus.

### MH-protein localizes to nuclear speckles in interphase nuclei

To better understand the nuclear fusion phenotype in *mh* embryos, we identified CG*9203* as the gene causing female sterility, as described below. The same gene was independently isolated by the Loppin group using a different strategy (Delabaere et al., 2014). We mapped the *mh* mutation to a 52.2 kbp region on the X-chromosome by finding two overlapping duplications in the P[acman] BAC (bacterial artificial chromosome) collection (Venken et al., 2010) that could rescue female sterility of the *mh*^1^ allele. Upon genomic sequencing and complementation tests (see Materials and Methods for details), we selected *CG9203*, the *Drosophila* ortholog of human Spartan which is involved in DNA repair as a candidate gene (Centore et al., 2012; Ghosal et al., 2012; Juhasz et al., 2012). *CG9203* is expressed maternally in ovaries (Gelbart and Emmert, 2013) and contains five non-synonymous mutations, one of which involves a conserved methionine in the conserved Sprt domain. Similar to the BAC clones, a 4432 bp genomic fragment containing the 2421 bp ORF, 1553 bp upstream and 282 bp downstream regions of *CG9203* could rescue female sterility. Rescue of *mh*^1^ sterility using the P[acman] clones and genomic DNA was partial. Approximately 10% of embryos produced by *mh*^1^/*mh*^1^ females hatched into larvae (32 of 323 eggs laid by *mh*^1^/*mh*^1^; PBac{DC300}VK00033/+), compared to 0% for females without *CG9203*-containing transgenes. The rescued larvae developed into adults without discernible morphological abnormalities that gave rise to stable *mh*^1^ homozygous stocks. The genomic duplication was able to rescue haploidy. Of *mh*^1^;PBac{DC300}/+ fixed syncytial blastoderm embryos in meta- or anaphase, 84.6% (22/26) were diploid, while the remaining 15.4% were haploid. In contrast, 100% (9/9) of *mh*^1^ embryos were haploid. Among the 22 rescued diploid *mh*^1^ embryos, none contained polyploidy nuclei, supporting the notion that loss of *mh* is responsible for chromosome collisions.

**Fig. 6.**
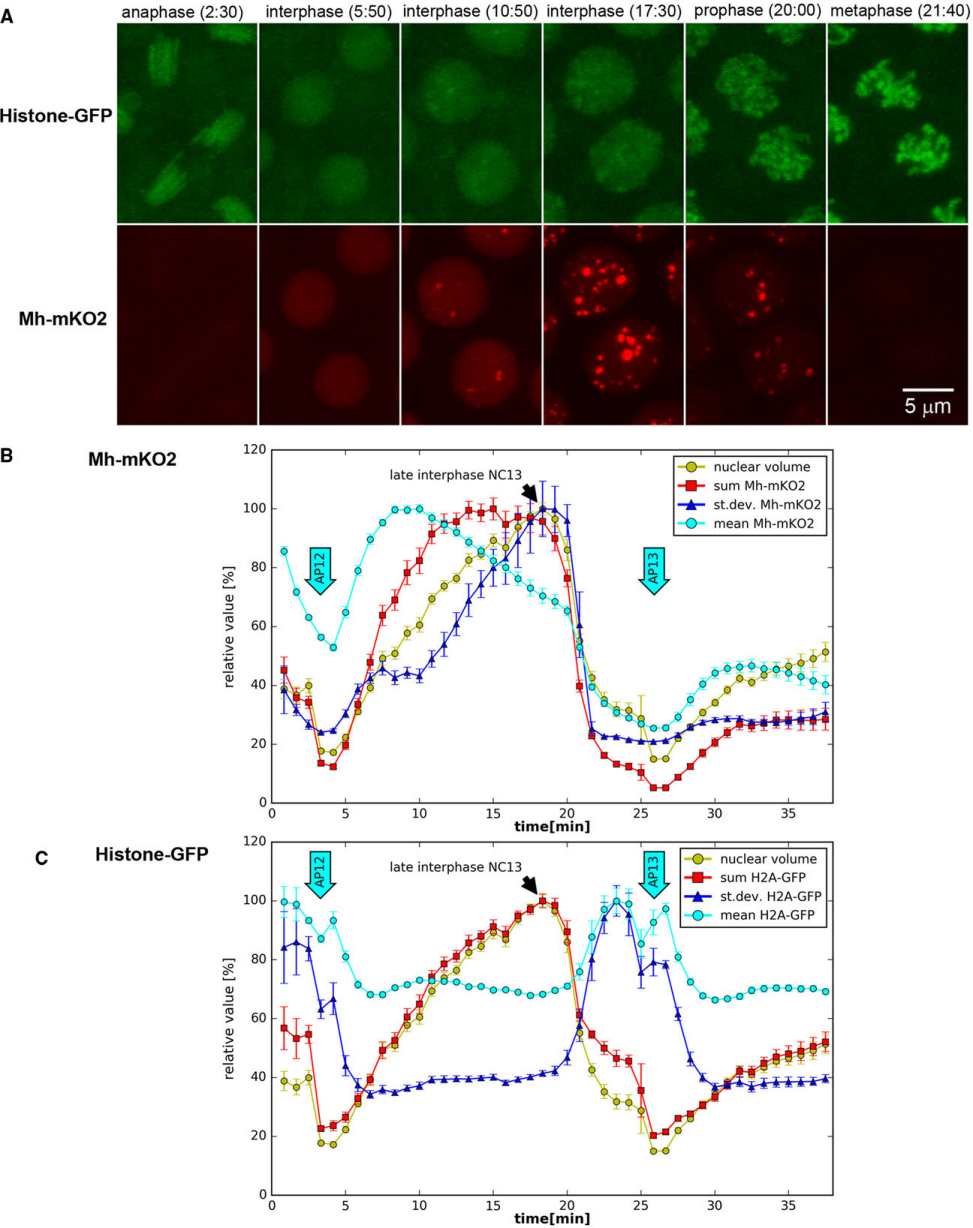
**Cell-cycle dependent changes of Mh-mKO2 localization.** (A) Mh-mKO2 (red) changes in intensity and localization during nuclear cycles of syncytial blastoderm embryos. Nuclear cycle phases were visualized using histone-GFP (green). Mh-mKO2 shows a uniform distribution in early interphase. As interphase progresses, Mh-mKO2 localizes to nuclear speckles that increase in number and brightness. The speckles are degraded during the progression from prophase to metaphase (see Movie 4). UASp-mh-mKO2 was expressed using the *nanos-Gal4:VP16* driver. Frames are MIPs of 3D stacks recorded using CLSM. (B) Fluctuations of Mh-mKO2 fluorescence in A between end of NC 12 and early interphase of NC14 were quantified in segmented nuclei. Mh-mKO2 intensity parameters (sum, mean, standard deviation) and nuclear volume are shown in relative units (mean±95% CI). Arrows labeled AP12/13 indicate anaphase of nuclear cycles 12 and 13. Speckle formation correlates with increased standard deviation. (C) The fluctuations of histone-GFP intensity are shown for comparison. *n*=4 (until metaphase 12), *n*=8 (AP12 to metaphase 13), *n*=16 (from AP13).

To study the sub-cellular localization of the Mh/CG9203 protein, we fused the *CG9203* ORF including five predicted introns in frame with the ORF of the orange fluorescent protein mKO2 (monomeric Kusabira Orange) and cloned it into the pUASp vector for maternal expression using the GAL4 system. Overexpression of *mh-mKO2* using the maternal *nanos-Gal4:VP16* driver rescued *mh*^1^ sterility and allowed establishment of a fertile stock, showing that the fusion protein retained Mh functionality. Co-expression of histone-GFP allowed us to study cell-cycle dependence of Mh-mKO2 localization in live embryos (Fig. 6; Movie 4). In early interphase, Mh-mKO2 accumulated in nuclei and showed uniform localization. As interphase progressed, the fusion protein showed concentration in speckles that increased in size and number. Mh-mKO2 clustering in the speckles began after 3-4 min of interphase, suggesting that speckle formation may correlate with the end of DNA replication. Localization to nuclear speckles was also reported for human Spartan (Machida et al., 2012). Once nuclei entered mitosis, the fusion protein was rapidly depleted and did not co-localize with condensed chromosomes. Therefore, the localization in mitosis does not provide a clue on how *mh* deficiency may contribute to chromosome fusion during metaphase and anaphase.

**Fig. 7.**
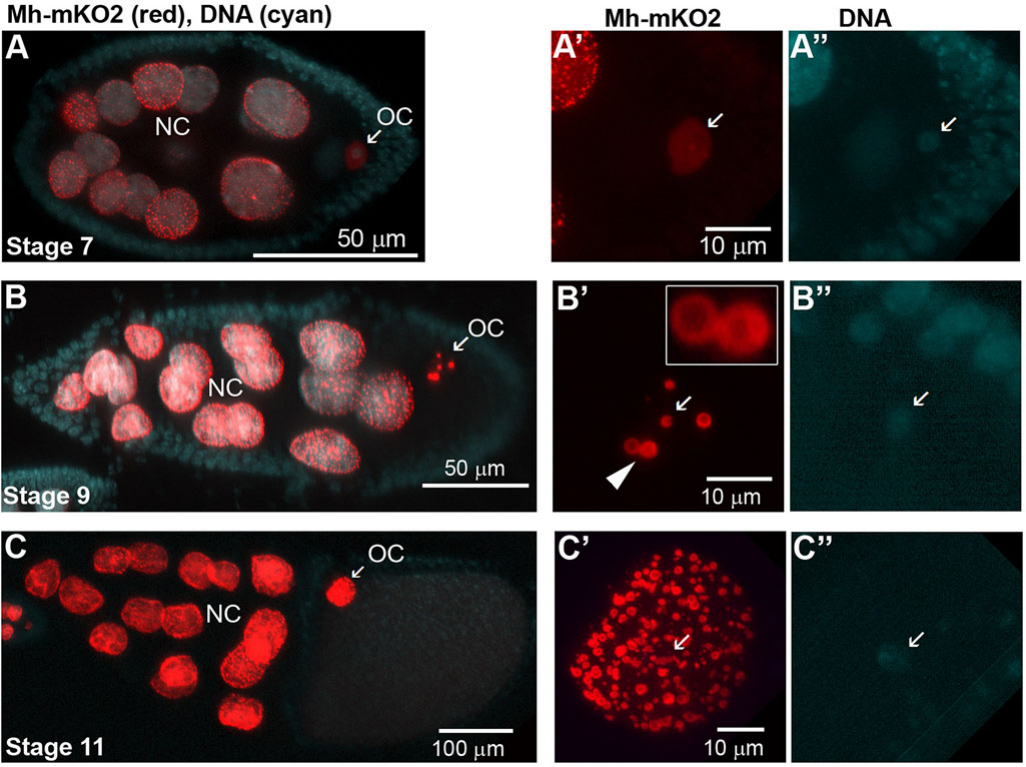
**Localization of Mh-mKO2 in the oocyte nucleus during oogenesis.** (A-C) Mh-mKO2 (red) expressed using maternal *nanos-Gal4:VP16* driver localizes to nuclear speckles in nurse cells (NC) and oocytes (OC). DNA (cyan) was stained with Hoechst dye. Panels to the right show separate Mh-mKO2 (A′-C′) and DNA staining (A″-C″) of the oocyte nuclei at higher magnifications. Arrows in A′-C′ indicate the positions of Hoechst-stained DNA in A″-C″. (A′) In early stages of oogenesis, Mh-mKO2 shows a uniform distribution in the oocyte nucleus. (B′) As egg chambers grow (stage 9), Mh-mKO2 clusters in six nuclear bodies, some of which display a doughnut-morphology (arrowhead, inset). (C′) In later stages, the quantity of speckles increases dramatically coinciding with an over 100-fold increase of size nuclear size compared to stage 7. Ovaries were dissected in PBS and fixed in 4% formaldehyde in PBS.

In adult ovaries, the localization of overexpressed MH-mKO2 showed developmental changes in oocytes (Fig. 7), while a speckled pattern was observed in nurse cells of all stages. In oocytes of early stages, Mh-mKO2 was distributed diffusely throughout the nucleus (Fig. 7A). The intensity of the fusion protein was higher in non-chromosomal region of the nucleus. As oocytes grew, Mh-mKO2 became concentrated to 5-8 speckles of different sizes that did not co-localize with DNA staining (Fig. 7B). The largest speckles were doughnut-shaped with diameters of up to 2.5 microns (Fig. 7B, inset). The quantity of nuclear speckles increased dramatically with egg chamber maturation and growth of the oocyte nucleus (Fig. 7C). When ectopically expressed in larval salivary glands, Mh-mKO2 showed distribution to speckles that did not co-localize with polytene chromosomes, further supporting the notion that protein does not have a strong affinity to chromatin (Fig. S11A). Intriguingly, the germline *UASp-mh-mKO2* and somatic *UAS-mh-mKO2* Gal4 effectors showed expression in larval and pupal testes (but not ovaries) without the presence of a Gal4 driver (Fig. S11B). Meanwhile, driverless fluorescence did not occur using other mKO2 fusion constructs, suggesting that the genomic *mh* fragment, possibly the introns, contain regulatory sequences activated in the male germline. Previous studies correlated speckle formation with DNA damage repair. We show that it also occurs in response to nuclear growth in development and cell cycle.

## DISCUSSION

### Quantitative microscopy of mitotic division cycles in embryogenesis

We developed a modular image analysis system for 3D time-lapse microscopy and applied it to the phenotypic characterization of the *maternal haploid* mutation in *Drosophila* embryos. Tracking and volume measurements demonstrated that nuclei of different ploidies in *mh* embryos could be traced back to collisions of mitotic chromosomes during the syncytial blastoderm stage. Nuclear collisions happened at metaphase or anaphase and involved chromosomes of different nuclei. We did not encounter a single case where the failure of sister chromatid segregation lead to increased ploidy. Fusions were observed at nuclear densities seen in wild-type embryos during nuclear cycles 12 and 13, ruling out the possibility that nuclear fusions resulted from crowding of nuclei. A previous study showed that haploid nuclei from *mh* donor embryos injected into diploid host embryos could give rise to haploid as well as diploid cell clones in adult flies (Santamaria, 1983). Our results provide an explanation for these observations.

The mechanism of nuclear fusion and its relationship to the *mh* gene function remain to be elucidated. Spartan, the homolog of the *mh* gene product is implicated in translesion synthesis, a system that helps replication forks bypass sites of DNA damage (Delabaere et al., 2014). As such, *mh* deficiency may lead to incomplete replication of the paternal pronucleus and its elimination from the zygote. Four models may explain chromosome collisions in syncytial embryos. First, loss of *mh* may affect the formation of actin-containing metaphase furrow that shields neighboring spindles from colliding in the absence of plasma membranes (Rikhy et al., 2015). Second, nuclear fusions in Centrosomin­-deficient embryos were attributed to centrosomal malfunction and reduced spacing of mitotic spindles (Megraw et al., 1999). Similarly, *mh*^1^ embryos may be more prone to centrosomal or spindles malfunction. Third, the fusion phenotype may be an indirect consequence of losing the paternal genome during fertilization, which may affect the formation of the metaphase furrow or centrosome function. Fourth, a second mutation on the *mh*^1^ chromosome, such as the female sterile mutation *fs(1)RK4* (Loppin et al., 2001), could be responsible for the phenotype. However, we did not find polyploid nuclei in *mh*^1^ homozygous embryos whose haploidy was rescued by a duplication of the *mh* gene, suggesting that haploidy and chromosome collisions are linked. Future research should focus on possible replication-independent functions since Mh-mKO2 accumulated in non-chromosomal nuclear speckles in late interphase, presumably after completion of replication. One known function of nuclear speckles is to serve as storage sites of RNA splicing factors (Lamond and Spector, 2003). Interestingly, a link between chromosome instability and localization to nuclear speckles was found for poly(ADP-ribose) polymerase-1 (PARP-1), which localizes to nuclear speckles in mouse oocytes and predisposes female gametes to genome instability (Yang et al., 2009).

### Nuclear size control

**Fig. 8.**
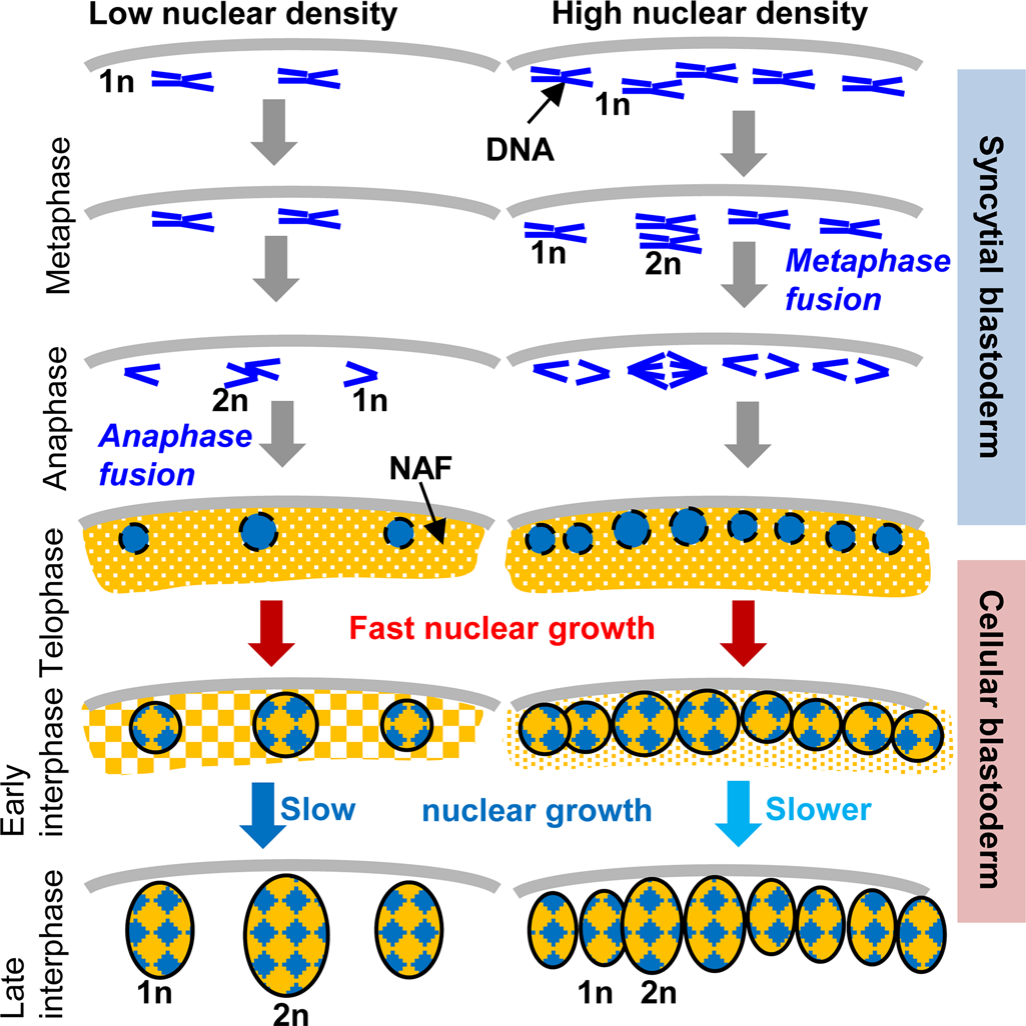
**Nuclear growth during cellularization is controlled by both DNA content and nuclear-to-cytoplasmic ratio.** *mh* embryos display variations in nuclear density and ploidy. During the syncytial blastoderm, ploidy changes result from collisions of mitotic chromosomes. In early interphase of the cellular blastoderm, rapid nuclear volume expansion depends mainly on ploidy (1n=haploid, 2n=diploid), suggesting that chromatin associated components recruit nuclear assembly factors (NAFs) from the cytoplasm. As NAFs become depleted in late interphase, nuclear growth slows down. Due to competition for a limited supply of cytoplasmic NAFs, nuclear growth displays an inverse correlation with nuclear density.

Taking advantage of the fact that we could track synchronized nuclei of different karyotypes in the same cytoplasm, we investigated the control of nuclear size in the context of *Drosophila* embryogenesis. Two opposing models explain the size of nuclei. While the evolutionarily conserved scaling of nuclear volume with genome size supports the intuitive model that DNA content is the main determinant of nuclear size, more recent evidence from various experimental systems favors the idea that cytoplasmic volume and components dictate nuclear volume (Edens et al., 2013). Our data show that both theories can be reconciled (Fig. 8). During mitosis, condensed chromatin occupies a space that is proportional to ploidy. In early interphase after exit from mitosis, nuclear volumes expand rapidly and growth rates and absolute sizes remain correlated with ploidy, indicating that some nuclear assembly factors are associated with chromatin. As interphase progresses, nuclear growth slows and crowding exerts a negative effect on nuclear growth, indicating that nuclei share a finite amount of other nuclear assembly factors that are deposited maternally into the egg chambers. As a result, nuclear volumes of the same karyotype can diverge between embryos. The conclusion that finite supplies of cytosolic factors are recruited for nuclear assembly are consistent with the ‘limited flat membrane hypothesis’ that proposes that growth of the nucleus is limited by the amount of ER membranes available in the cytoplasm for incorporation into the NE (Webster et al., 2009).

Based on our experiments, the variations of nuclear size and growth in *mh* embryos can be explained by variations in ploidy and nuclear density. However, to fully rule out genetic effects of the *mh* mutation and strengthen the argument of generality, we will need to extend our analysis to other genetic perturbations. One example is the mutant *daughterless-abo-like* (*dal*) where embryos, due to nuclear division defects, enter cellularization at half the nuclear density of wild-type embryos (Sullivan et al., 1990). Although no quantification was carried out, nuclei and cells were reported to be much larger than in wild-type embryos, indicating that the inverse correlation between nuclear size and density in *mh* embryos may reflect a general response of nuclei to increased cytoplasmic volume. While the literature reports that *mh* embryos undergo 14 nuclear cycles, live imaging helped to uncover that a subset of embryos undergo their final synchronized mitosis at nuclear densities similar to wild-type embryos in nuclear cycles 12 or 13. Since nuclear density depends on the number of division cycles and the depletion of nuclei, a key experiment would be to perform live imaging of all nuclear cycles from the zygote onwards. However, the single-photon confocal laser scanning microscopy (CLSM) used in this study has limited depth penetration and could only image the cortical cycles 10-13. Multi-photon confocal microscopy or light sheet microscopy (Keller et al., 2010) appear suitable to overcome this optical limitation and record nuclear cycles 1-9 in the interior of the egg.

## MATERIALS AND METHODS

### *Drosophila* stocks

Nuclei and mitotic chromosomes in live *maternal haploid* (*mh*^1^) and control *Drosophila* embryos were visualized using the *His2Av-GFP* reporter gene on the third chromosome that expresses Histone-H2Av fused to green fluorescent protein (GFP) (Clarkson and Saint, 1999). The reporter gene was combined with the X-chromosome *y*^1^ *w*^a^ *mh*^1^/*FM7a* (Bloomington *Drosophila* Stock Center; B-7130). Fly stocks were obtained from the Bloomington Drosophila Stock Center. Female sterility associated with *mh*^1^ could be rescued with two overlapping BAC X-chromosomal duplications X: 15453241-15544202 and X: 15397371-15505482 of the stocks *w*^1118^; *Dp(1;3), PBac{DC300}VK00033* (B-30414) and *w1118*; *Dp(1;3), PBac{DC521}VK00033* (B-32322), respectively. The *nanos-Gal4:VP16* driver (B-4937) was used for maternal overexpression, *Smid-Gal4* (B-27893) for expression in salivary glands. P-element mediated germline transformation was carried out by an external service provider (BestGene, Chino Hill, CA). Stocks newly generated for this study were deposited at Vienna *Drosophila* Resource Center (VDRC): http://stockcenter.vdrc.at.

### Molecular biology and identification of the *mh* gene

Genomic DNA of *mh*^1^ homozyogous adults was isolated using the Wizard Genomic DNA purification kit (Promega). Genomic DNA of the mutant X-chromosome was re-sequenced by a service provider (BGI, Shenzhen, China). To rescue female sterility linked to the *mh*^1^ chromosome, we amplified by PCR 4432 bp of genomic DNA from wild-type Canton-S flies containing the *CG9203* gene. The genomic DNA fragment (X: 15,476,953-15,472,522) contained the 2432 bp ORF with five exons and four introns, a 1637 bp upstream and 282 bp downstream region. The PCR primers included *Xba*I and *Xho*I restriction enzyme (RE) sites that were used to insert the genomic fragment into the *pCasper4* transformation vector. To study protein localization of the *CG9203* gene product, the *CG9203* ORF was fused in frame with the cDNA of monomeric Kusabira Orange 2 (mKO2) (Sakaue-Sawano et al., 2008) and cloned into *pUASp* and *pUAST* derived expression vectors using the Gateway recombination system (Invitrogen) as previously described (Puah and Wasser, 2016). In short, the *CG9203* ORF was amplified by PCR disabling the predicted stop codon and cloned directionally by topoisomerase I to create a Gateway entry vector. Recombination between entry clone and the pUASp-GC-mKO2/pUAST-GC-mKO2 destination vectors created the pUASp-gc9203-mKO2 and pUAST-cg9203-mKO2 P-element germline transformation vectors.

### Time-lapse microscopy

Sample preparation and *in vivo* imaging were performed as previously described (Chinta and Wasser, 2012). Embryos at 0 to 2 h old were dechorionated for 2 min with a 50% bleach solution, washed with PBS, transferred to an uncoated 32 mm glass bottom dish (MatTek, Ashland, MA) and covered with a solidified layer of 0.8% low melting point agarose in PBS to prevent dehydration during imaging. Time-lapse imaging was carried out at room temperature (21-23° C) using a high-speed, line-scanning Zeiss 5 Live confocal laser scanning inverted microscope (Carl Zeiss, Jena, Germany) and a 63x/1.4 oil DIC Plan-Apochromat objective. For single-channel imaging of histone-GFP, we used an excitation wavelength of 489 nm at 0.5% to 1% laser power (maximum output 100 mW). We selected a 505 nm emission long pass filter and set the slit size to 10 µm. The optimal spacing between z slices of 0.44 µm was suggested by the manufacturer's acquisition software. We recorded the 3D image stacks, consisting of 66-70 slices of 1024×1024 pixels (zoom 1.0, 8 bits), at 50 to 60 s intervals for up to 4 h. Image acquisition focused on the head region to limit the number of cells migrating out of the field of view due to gastrulation. The voxel dimensions in x, y and z were 0.1×0.1×0.44 µm. Dual-channel recording of histone-GFP and Mh-mKO2 was carried out at 50-second intervals using 488 nm and 532 nm lasers at 2.8% power (band pass filter NFT 535, long pass filter 550 nm). 3D image stacks contained 37 optical sections with voxel dimensions of 0.1×0.1×0.71 µm (x,y,z). The imaging setup did not result in photo-toxicity as control embryos developed to become larvae. Images were saved as LSM files using the Zeiss acquisition software.

### Image analysis pipeline

Our image analysis pipeline consisted of five main steps: (A) image data reorganization, (B) 3D image segmentation, (C) optional post-processing of segmentation outputs, (D) time-series analysis, and (E) optional 3D visualization (Fig. 1). (A) 3D time-lapse imaging generated sizeable datasets of 180-240 time points which were saved as LSM files containing between 13 to 18 gigabytes (GB) of data. To facilitate downstream processing, we fragmented the 4D CLSM datasets into portions of 10 time points and saved them as files of the open source image cytometry standard (ICS) format (Dean et al., 1990) using our custom-built TLM-converter software (Puah et al., 2011). The newly reported tools of the pipeline (C-E) were implemented in C++ using the Open CV computer vision library (http://opencv.org/), the libics v1.5 reference library (http://libics.sourceforge.net/) for reading and writing multi-dimensional ICS files and the Visualization tool kit (Vtk) (Schroeder et al., 2006). The graphical user interfaces were implemented in the .NET (Microsoft) environment. Software, manuals and samples datasets are available for download at following link: https://figshare.com/s/e030e8dec869d8095f53.

### 3D image segmentation

To detect and extract features of interphase nuclei and mitotic chromosomes, we applied a fully automated 3D image segmentation tool (Chinta and Wasser, 2012). The segmentation method is based on multiple level sets (MLS) and adapts to differences in intensity, shape and size as chromosomes progress through the cell cycle phases. We used the batch processing module of our tool to segment up to eight datasets, containing 10 time points each, in parallel. On a desktop PC with an Intel i7 core CPU, 80 time points were processed in approximately 2 h. We selected the same parameters for all 4D datasets used in this study: Δk0=0.5, ΔkM=0.5 and ΔkRG=0.2 are tuning factors for the surface evolution during the object detection, seed detection and region growing steps, respectively. Minimum seed size was set to 1 µm^3^ and minimum detected object size to 5 µm^3^ to filter out non-nuclear objects. The Batch 3D MLS segmentation tool exports four output files per image stack (Fig. S1). (1) The master segmentation project file (*.SGPJ) stores information about input images, segmentation parameters and the names of three associated output files, which contain the contours and features of detected objects. (2) The segmented objects file (.CSV format) contains a table of detected 3D objects and their features, including a unique object ID, centroid coordinates, shape and intensity parameters. (3) 3D surface files (.cont) store 3D ROIs as sets of overlapping 2D contours that can be used to restore and modify segmentation projects during the post-processing step. (4) Silhouette contour files (.cont) contain one silhouette contour per 3D object. Silhouette contours are extracted from the projection of all polygons belonging to a 3D object and are used to evaluate the boundaries of detected objects in MIP images.

### Segmentation post-processing

As an optional step in the image analysis pipeline, the post-processing module extracts additional features from segmentation outputs for the following purposes. (1) It computes additional shape, intensity and texture features that are not required for all applications. Here, we obtained Mh-mKO2 intensity parameters within the regions of histone-GFP labeled nuclei (Fig. 6B,C). In a previous study, we obtained 3D shape and texture features required for cell cycle classification (Du et al., 2011). (2) It can save cropped 3D regions of 3D stacks for iso-surface rendering. (3) It helps to separate merged nuclei, a segmentation error particularly affecting image stacks of the early cellular blastoderm stage. We implemented a simple 3D shape-split algorithm that was applied to all 2D polygons of 3D objects within a user-defined range of volumes (default 10 to 1000 µm^3^). The algorithm finds the shortest cutting line between two contour points that fulfills three constraints (Fig. S3): (1) a minimum area of the input polygon (default 700 pixels or 7 µm^2^), (2) minimum perimeter of the smaller output polygon (default 70 pixels or 7 µm) and (3) a maximum ratio between cutting distance and the perimeter of smaller output polygon (default 0.3). If any of the constraints are violated, no splitting occurs. Splitting is repeated until no input polygon is left that meets all constraints. 3D objects are reconstructed from cut polygons based on a minimum overlap between polygons in consecutive focal planes (default 50% with regards to the smaller polygon).

### Time-series analysis

Time-series analysis, such as tracking nuclei and quantifying morphological changes during development, was performed using the einSTA (integration and editing of Segmentation, Tracking and Annotation) custom software. einSTA saves computational resources by only using processed data without the need to load large multi-dimensional image data. The workflow of constructing and quantifying tracks of proliferating nuclei consists of four main steps.

#### Edit initial tracking project file

The user constructs an initial tracking project file (.TRPJ) using a text editor (Fig. S1). The header lists file type (here ‘Tracking-Project 2’), number of frames, image dimensions, and the names and types (integer, double, text) of features. The current prototype uses up to 98 shape, intensity and texture features to describe biological objects. The header is followed by a list of MIP images (1 per frame). The third mandatory component is a list of segmented object files. Each row corresponds to a single frame and can contain one or more comma separated ROI files. This way, quantification can be performed with ROIs derived from the outputs of different segmentation methods.

#### Create composite segmentations

Biological objects, such as chromosomal DNA in different cell cycle phases, can be heterogeneous in terms of intensity, shape, texture and location, resulting in variable segmentation quality within the same image stack. To improve segmentation quality, we introduced the concept of composite segmentation. The composite segmentation contains a combination of ROIs derived from different segmentation outputs. We evaluated segmentation quality using silhouette contours and centroids drawn on MIP images (Fig. S2). Initially, we populated the composite with all ROIs from the most accurate segmentation. Later, errors such as fused, split or missing objects were rectified by a range of editing functions. In this study, we created a second layer of ROIs by applying a shape split method in the post-processing module to the original segmentation (Fig. S3, see above).

#### Tracking of nuclei

Tracking creates associations between objects in subsequent frames and was performed in a semi-automated fashion. An automated bilateral nearest neighbor search was combined with manual error correction. The nearest neighbor search worked well during interphase when displacements were small (Fig. S4A). However, this approach failed during anaphase of mitosis when mean displacements could be as high as 15 µm microns per 55 s (Fig. S4C). A set of visualization techniques, such as overlaying subsequent frames and displaying links between centroid (Fig. S4A-C), helped to validate tracks. Missing or wrong links were edited manually. Selected tracks and their features were exported as comma separated text files for further statistical analysis.

#### Quantification of nuclear densities

To monitor the proliferation and elimination of nuclei on the cortex of embryos, we used the x and y components of the centroid coordinates as inputs to determine convex hulls using the cvConvexHull2 function of the OpenCV library. Dividing the number of ROIs by the hull area produced an estimate of nuclear density. Prior to calculating the convex hull, we applied a set of constraints such as ranges for volume (10 to 1000 µm^3^) and depth (0 to 25 µm). Nuclear hull statistics were exported as flat files for further statistical analysis.

### Dynamic iso-surface visualization (E)

We developed the custom tool DyVis3D (dynamic visualization in 3D) to generate and view interactive animations of selected tracks using three steps. (1) Tracks of nuclei created in einSTA are exported as flat files. (2) The post-processing module imports these tracks to extract foreground regions of 3D ROIs within their bounding boxes, while background regions are masked in black. Cropped image stacks are saved along with a time-lapse 3D (.TL3D) file describing the tracks. (3) In DyVis3D, the TL3D file is opened to reconstruct a dynamic scene composed of wireframe models representing the nuclei. The track information enables the selective rendering (color, transparency, level of detail, style) of individual tracks. The optimal thresholds obtained during the MLS surface evolution were used as initial intensity thresholds for iso-surface extraction.

### Statistical analysis

Statistical data analysis was performed using Excel (Microsoft), Minitab 16 (Minitab Inc.), R statistical software (R Core Team, 2013) and Python (Python Software Foundation). R was used to compute Student's *t*-tests, confidence intervals of means and proportions. Minitab was applied to produce interval and box-and-whisker plots (boxplots). Excel was applied to calculate basic statistics, perform regression analysis and plot line and bar charts. The Python library matplotlib was used to generate some of the 2D plots (Fig. 6B,C).
